# Effects of Traditional Chinese Patent Medicine on Essential Hypertension: A Systematic Review

**DOI:** 10.1097/MD.0000000000000442

**Published:** 2015-02-06

**Authors:** Xingjiang Xiong, Pengqian Wang, Yuqing Zhang, Xiaoke Li

**Affiliations:** From the Department of Cardiology, Guang’anmen Hospital (XX); Institute of Basic Research in Clinical Medicine, China Academy of Chinese Medical Sciences, Beijing, China (PW); Department of Clinical Epidemiology and Biostatistics, McMaster University, Hamilton, Ontario, Canada (YZ); and Bio-organic and Natural Products Laboratory, McLean Hospital, Harvard Medical School, Belmont, Massachusetts, USA (XL).

## Abstract

Traditional Chinese patent medicine (TCPM) is widely used for essential hypertension (EH) in China. However, there is no critically appraised evidence, such as systematic reviews or meta-analyses, regarding the potential benefits and disadvantages of TCPM to justify their clinical use and recommendation. The aim of this review was to systematically evaluate and meta-analyze the effects of TCPM for EH.

Seven databases, the Cochrane Library, PubMed, EMBASE, the China National Knowledge Infrastructure, the Chinese Scientific Journal Database, the Chinese Biomedical Literature Database, and the Wanfang Database, were searched from their inception to August 2014 for relevant studies that compared one TCPM plus antihypertensive drugs versus antihypertensive drugs alone. The methodological quality of the included trials was assessed using the Cochrane risk-of-bias tool. The primary outcome measures were mortality or progression to severe complications and adverse events. The secondary outcome measures were blood pressure (BP) and quality of life (QOL).

Seventy-three trials, which included 8138 patients, on 17 TCPMs were included. In general, the methodological quality was low. Two trials evaluated the effects of TCPMs on mortality and the progression to severe complications after treatment, and no significant difference was identified compared with antihypertensive drugs alone. No severe adverse events were reported. Thirteen TCPMs used in complementary therapy significantly decreased systolic BP by 3.94 to 13.50 mmHg and diastolic BP by 2.28 to 11.25 mmHg. QOL was significantly improved by TCPM plus antihypertensive drugs compared with antihypertensive drugs alone.

This systematic review provided the first classification of clinical evidence for the effectiveness of TCPM for EH. The usage of TCPMs for EH was supported by evidence of class level III. As a result of the methodological drawbacks of the included studies, more rigorously designed randomized controlled trials that focus on mortality and cardiovascular events during long-term follow-up are warranted before TCPM can be recommended for hypertensive patients. Two TCPMs, Song ling xue mai kang capsules and Yang xue qing nao granules, should be prioritized for further research.

## INTRODUCTION

Hypertension is one of the most important worldwide public-health challenges because of its high frequency and concomitant risks of cardiovascular and kidney disease.^1,2^ Across all World Health Organization regions, approximately 62% of strokes and 49% of myocardial infarctions are caused by high blood pressure (BP).^3^ Hypertension has been identified as the fourth leading cause of the global burden of disease.^4^ The prevention and effective treatment of essential hypertension (EH) is of utmost importance both in China and the West.^5^ However, the effective control of hypertension is far from satisfactory and is limited by the availability, cost, and adverse effects of antihypertensive medications.^1^ Therefore, a certain proportion of patients have turned to complementary and alternative medicine (CAM), including traditional Chinese medicine (TCM), in the search for a treatment modality with potential efficacy and few adverse effects. In Western countries, interest in TCM stems from the hope that it might complement Western medicine. This situation is also partially supported by recent studies.^6^

The most significant distinction between China and the West in EH treatment is the application of acupuncture and traditional Chinese patent medicines (TCPMs), which have been considered complementary or adjunctive therapies for symptom management and quality of life (QOL) enhancement along with mainstream care.^7–9^ Although no TCPMs have been listed as an investigational new drug by the US Food and Drug Administration, all TCPMs have resulted in great economic and clinical benefits in China. For example, yang xue qing nao granules are composed of Rehmannia (Dihuang, Radix Rehmanniae Glutinosae), Chinese Angelica Root (Danggui, Radix Angelicae Sinensis), Gambir Vine Stems and Thorns (Gouteng, Ramulus Uncariae Cum Uncis), Mother of Pearl (Zhenzhumu, Concha Margaratiferae), Foetid Cassia Seeds (Juemingzi, Semen Cassiae Torae), Prunella (Xiakucao, Spica Prunellae Vulgaris), White Peony Root (Baishao, Radix Albus Paeoniae Lactiflorae), Szechuan Lovage Root (Chuanxiong, Rhizoma Ligustici Chuanxiong), Spatholobus (Jixueteng, Caulis Milletiae Seu Spatholobi), Corydalis Rhizome (Yanhusuo, Corydalis Rhizome), and Chinese Wild Ginger (Xixin, Herba Asari Cum Radice). Yang xue qing nao granules have been extensively tested in various types of clinical trials. In 2012, the global market of yang xue qing nao granules was approximately 0.5 billion RMB (equivalent to 81.7 million USD). Several randomized controlled trials (RCTs) and systematic reviews regarding the efficacy of acupuncture for EH have been published in English.^10–12^ However, there is insufficient clinical evidence to support or discourage the use of many commonly used TCPMs in hypertensive patients,^13–14^ especially in conjunction with conventional therapy, despite wide acceptance and authoritative recommendations by the China Food and Drug Administration (CFDA) (available at http://www.sda.gov.cn) and the Pharmacopoeia of the People's Republic of China (2010 edition) for EH in China.^15^ Therefore, confirmation of the effectiveness of TCPMs as complementary therapies could have a substantial impact on EH management worldwide.

These TCPMs are composed of >100 herbs, most of which have specific antihypertensive effects both *in vitro* and *in vivo* when tested alone. Pharmacological studies have indicated that most studied TCPMs can be used to lower BP, and the potential mechanisms may be related to improvements in the plasma levels of endothelin, calcitonin gene-related peptide, and nitric oxide; the inhibition of sympathetic activity; and the regulation of the rennin-angiotensin system.^16–19^ The most commonly used formulations of TCPM for EH include tablets, capsules, pills, granules, and oral liquid. It remains unknown whether there is robust evidence on the clinical effects of TCPM or whether TCPM can be recommended for routine treatment. However, some relevant trials have been reported in China. Thus, the objective of this study was to provide a comprehensive systematic review to summarize and evaluate the evidence regarding the efficacy of TCPM combined with antihypertensive drugs as a complementary therapy for hypertension. To our knowledge, this is the first systematic English-language review of the clinical trials of all TCPMs for the treatment of EH.

## METHODS

A methodological evaluation and meta-analysis were conducted in accordance with the recommendations of the Cochrane Collaboration.

### Eligibility Criteria

#### Type of Studies

Only RCTs were eligible for this review, with no restriction on language or publication status. According to the methodological quality of the included RCTs, they were categorized into 3 levels: trials with a clear method of randomization were defined as definite RCTs; trials with an unknown methodology, in which the claimed “RCTs” may not be real “RCTs,” were defined as potential RCTs; and trials that included treatment and control groups, and in which treatment allocation was not necessarily randomized, were defined as controlled clinical trials (CCTs).

#### Types of Participants

Participants enrolled in the studies of this review had to meet at least one of the current or past guidelines or definitions of EH, which are described as follows: systolic BP (SBP) ≥140 mmHg and/or diastolic BP (DBP) ≥90 mmHg on at least 2 occasions with off-antihypertensive treatment (after a 4-week washout period) and/or treatment with antihypertensive medication, while excluding secondary hypertension.^1^ Trials without a description of the detailed diagnostic criteria but that included patients with definite EH were also included. Because there are many TCM syndromes (also known as zheng or patterns) of EH,^7,8,9,13,14^ no restriction regarding the diagnostic criteria of TCM was utilized. Trials that included patients of any age, sex, or ethnic origin with EH were eligible. There were no restrictions on population characteristics.

#### Types of Interventions

TCPMs that had been subjected to a relatively strict drug evaluation process, including active constituent identification, a compatibility mechanism study, efficiency and safety evaluation, and RCTs were included. Substantial systemic preclinical or clinical data (regardless of whether they were provided in publications) were available for these selected TCPMs. In our review, only TCPMs listed in the Chinese National Essential Drug (CNED) of 2012 (available at http://www.sda.gov.cn), the Pharmacopoeia of the People's Republic of China (PPRC) (2010 edition), and commonly used TCPMs in current clinical practice for EH were included in the analysis. Drugs in CNED and PPRC represent the most important therapies listed by the Chinese government, which have been studied extensively and proven to be safe and effective in treatment. More clinical studies reported the use of commonly used TCPMs. In total, 82 TCPMs were identified for further evaluation.

All parallel RCTs of the 82 TCPMs that compared 1 TCPM plus antihypertensive drugs (TPAD) versus antihypertensive drugs alone for EH were eligible. Co-interventions were matched in both the treatment and control groups. Trials were excluded for the following reasons: a multimodal intervention, including TCPM and other complementary and alternative approaches, was used in either the treatment or control groups; a combination of >2 TCPMs was included in the treatment group; head-to-head comparisons of different types of TCPMs were utilized without a standard Western medicine control group; the types and dosages of antihypertensive drugs used in the treatment or control groups were not the same; only biochemical markers, rather than BP, were reported; no data on BP outcome could be extracted; and duplicated publications reported the same results. Trials that evaluated any TCPM formulation (tablets, capsules, or pills) were included regardless of the treatment period length and the treatment dosage.

#### Types of Outcome Measures

For inclusion, RCTs had to have evaluated at least one of the following outcomes. The primary outcome measures included mortality, progression to severe complications, and adverse events (AEs). Severe complications were defined as coronary heart disease, myocardial infarction, heart failure, cerebral infarction, cerebral hemorrhage, transient ischemic attack, renal failure, and retinopathy. The secondary outcome measures included SBP, DBP, and QOL after treatment. Trials were excluded if missing information about the outcome measure were found.

### Search Strategy

Studies on 82 TCPMs were identified through searches of the following 7 electronic databases from their inception until August 17^th^, 2014: the Cochrane Library (searched in August 2014), PubMed (1959–2014), EMBASE (1980–2014), the China National Knowledge Infrastructure (1979–2014), the Chinese Scientific Journal Database (1989–2014), the Chinese Biomedical Literature Database (1978–2014), and the Wanfang Database (1985–2014). Because TCPMs were invented and are primarily used in China, we conducted a literature search in the 4 main Chinese electronic databases to include the maximum possible number of clinical trials. To include unpublished studies, we also searched the website of the Chinese clinical trial registry (available at http://www.chictr.org/) and the international clinical trial registry maintained by the US National Institutes of Health (available at http://clinicaltrials.gov/).

For the database searches, the following keywords were used in combination: (“clinical trial” OR “randomized trial” OR “randomized controlled trial” OR “randomised controlled trial”) AND (“essential hypertension” OR “primary hypertension” OR “hypertension” OR “high blood pressure” OR “blood pressure”) AND (“traditional Chinese patent medicine” OR “Chinese patent medicine” OR “song ling xue mai kang capsule” OR “Pinus armandi Franch benefiting blood vessel capsule” OR “yang xue qing nao granule” OR “nourishing the blood and clearing brain granule” OR “liu wei dihuang pill” OR “liu wei dihuang wan” OR “rehmannia six formula” OR “quan tianma capsule” OR “total gastrodia capsule” OR “xin ke shu” OR “smoothing heart capsule” OR “qing nao jiang ya tablet” OR “clearing brain and antihypertensive tablet” OR “tianma gouteng granule” OR “granule of gastrodia and uncaria” OR “qiang li tianma duzhong capsule” OR “powerful gastrodia and eucommia capsule” OR “qi ju dihuang pill” OR “lycii, chrysanthemi and rehmanniae pill” OR “niuhuang jiang ya pill” OR “calculus bovis antihypertensive pill” OR “zhen ju jiang ya tablet” OR “pearl and lycii antihypertensive pill” OR “qiang li ding xuan tablet” OR “powerful fixing vertigo tablet” OR “tianma shouwu tablet” OR “gastrodia and polygonum multiflorum tablet” OR “qing gan jiang ya capsule” OR “clearing liver and antihypertensive capsule” OR “qing xuan jiang ya tablet” OR “clearing vertigo and antihypertensive tablet” OR “tian shu capsule” OR “capsule of chuanxiong rhizome and gastrodia tuber for headache” OR “lingyangjiao capsule” OR “cornu saigae tataricae capsule” OR “duzhong shuang jiang pao dai ji” OR “eucommia ulmoides bubble bag agent” OR “an gong jiang ya pill”OR “an gong antihypertensive pill” OR “yun tong ding tablet” OR “vertigo and headache fixing tablet” OR “fu fang luobuma granule” OR “compound apocynum granule” OR “xin nao jing tablet” OR “clearing heart and brain tablet” OR “zhen nao ning capsule” OR “calming brain capsule” OR “jiang ya ping tablet” OR “antihypertensive tablet” OR “shanzha jiang ya tablet” OR “hawthorn antihypertensive tablet” OR “xing nao jiang ya pill” OR “clearing brain and antihypertensive pill” OR “xuan yun ning granule” OR “fixing vertigo granule” OR “yu feng ning xin tablet” OR “calming wind and relieving heart tablet” OR “yi nao ning tablet” OR “reinforcing brain tablet” OR “yi ling jing mixture” OR “supplementing vital essence mixture” OR “yang yin jiang ya capsule” OR “nourishing yin and antihypertensive capsule” OR “huan jing jian oral liquid” OR “sperm-return oral liquor” OR “tian mu jiang ya tablet” OR “gastrodia and pearl antihypertensive tablet” OR “xia sang ju granule” OR “prunella, folium mori and chrysanthemum granule” OR “jiang ya bi feng tablet” OR “antihypertensive and avoiding wind tablet” OR “jiang ya granule” OR “antihypertensive granule” OR “jiang ya tablet” OR “antihypertensive tablet” OR “jiang ya pill” OR “antihypertensive pill” OR “fu fang duzhong tablet” OR “compound eucommia tablet” OR “duzhong ping ya capsule” OR “eucommia antihypertensive capsule” OR “shan lv cha jiang ya tablet” OR “hainan holly leaf antihypertensive tablet” OR “luobuma jiang ya tablet” OR “apocynum antihypertensive tablet” OR “shan zhuang jiang zhi tablet” OR “hawthorn lipid-lowering tablet” OR “luo huang jiang ya tablet” OR “apocynum and rhubarb antihypertensive tablet” OR “luo ji jiang ya tablet” OR “apocynum and stephaniae tetrandrae antihypertensive tablet” OR “zhen xin jiang ya tablet” OR “calming heart antihypertensive tablet” OR “ju ming jiang ya tablet” OR “chrysanthemum and cassia seed antihypertensive tablet” OR “fu fang lingyangjiao capsule” OR “compound cornu saigae tataricae capsule” OR “fu fang xiakucao jiang ya syrupus” OR “compound prunella antihypertensive syrupus” OR “gao xue ya su jiang pill” OR “quickly lowering blood pressure pill” OR “xue ya an ba bu gao” OR “antihypertensive babu plaster” OR “jiu qiang nao li qing” OR “enduring and powerful clearing brain pill” OR “luobuma tea” OR “apocynum tea” OR “luobumaye granule” OR “apocynum venetum leaves granule” OR “sanqihua granule” OR “notoginseng flower granule” OR “xin ling pill” OR “calming heart pill” OR “dong qing bu zhi” OR “holly fill juice” OR “duzhong granule” OR “eucommia granule” OR “feng shi xi tong tablet” OR “siegesbeckiae and clerodendroa trichotomum tablet” OR “shanzha jing jiang zhi tablet”OR “hawthorn lipid-lowering tablet” OR “guan tong tablet” OR “dredging coronary artery tablet” OR “xing nao niu huang qing xin tablet” OR “calculus bovis calming brain and heart tablet” OR “kang mai xin oral liquid” OR “benefiting blood vessel and heart oral liquid” OR “mai jun an tablet” OR “calming pulse tablet” OR “mai luo tong tablet” OR “promoting blood circulation tablet” OR “quan duzhong capsule” OR “total eucommia capsule” OR “yan you cha” OR “yan you tea” OR “su xiao niuhuang pill” OR “quick-actng calculus bovis pill” OR “qing re xing nao ling tablet” OR “clearing heat and calming brain tablet” OR “qing re ming mu tea” OR “clearing heat and improving eyesight tea” OR “rong shuan capsule” OR “thrombolysis capsule” OR “shan hua jing granule” OR “hawthorn and chrysanthemi granule” OR “fu fang tianma granule” OR “compound gastrodia granule” OR “shu luo tablet” OR “dredging collaterals tablet” OR “shu xin ning tablet” OR “regulating heart tablet” OR “xin shu bao tablet” OR “relieving heart tablet” OR “xin xue ning tablet” OR “calming heart and blood tablet” OR “xiatianwu injection” OR “corydalis injection” OR “xianlingpi granule” OR “epimedium granule” OR “xin an capsule” OR “calming heart capsule” OR “xin an ning tablet” OR “calming heart and tranquilizing mind tablet” OR “xin mai tong tablet” OR “promoting heart and blood circulation tablet”).

Furthermore, the reference lists of the identified original articles were manually searched for additional eligible studies. The pharmaceutical companies that manufacture TCPMs were contacted to identify further published and unpublished studies. The literature search was independently conducted by 2 reviewers, and disagreements were settled by discussion between the reviewers or a consultation with a third party.

### Data Extraction and Management

Two reviewers independently screened the trials based on the titles and abstracts. The following information was extracted from the eligible trials that met the inclusion criteria: basic characteristics (age, gender, race of participants, and sample size) of the included subjects and basic characteristics (authors, title of study, year of publication, randomization, allocation concealment, blinding, intention to treat analysis, drop-out or withdrawal, diagnosis standard, TCPM name, interventions in the treatment and control groups, outcome measures, treatment duration, and safety reporting) of the included trials:. Missing data were obtained from the original authors via email, fax, or telephone when possible. Disagreements were resolved by discussion with a third reviewer.

### Risk of Bias in Individual Studies

Two reviewers independently assessed the risk of bias in the included studies using tools from the Cochrane Handbook for Systematic Review of Interventions Version 5.1.0 (updated March 2011). The factors assessed included the following 7 items: random sequence generation (selection bias); allocation concealment (selection bias); blinding of participants and personnel (performance bias); blinding of outcome assessment (detection bias); incomplete outcome data (attrition bias); selective reporting (reporting bias); and other sources of bias (from Chapter 8: assessing risk of bias in included studies). For each item, the risk of bias was classified as “low,” “unclear,” or “high” according to the previously discussed criteria.

### Data Analysis

Data regarding outcomes in the eligible studies were combined in the meta-analysis using the Revman 5.1 software provided by the Cochrane Collaboration. Dichotomous data were presented as the risk ratio (RR), and continuous outcomes were presented as the weighted mean difference (WMD); both measurements were reported with the 95% confidence interval (CI). Statistical heterogeneity between the studies was significant when *I*^*2*^ ≥50%. Fixed-effects model was used if there was no significant heterogeneity of the data (*I*^*2*^ < 50%); random-effects model was used if significant heterogeneity existed. The risk of publication bias was assessed by funnel plot analysis for each meta-analysis if sufficient studies (ie, >10) were identified.^20^

## RESULTS

### Literature Search

**FIGURE 1:**
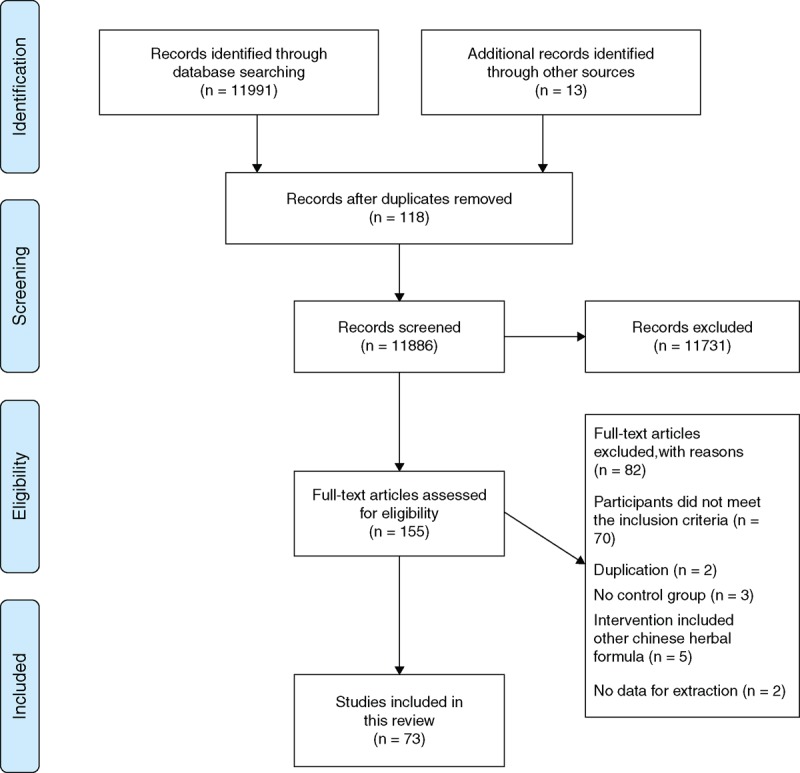
Flowchart of the study selection and identification process.

Figure 1 shows a flowchart that depicts the study search and selection process. An initial screening yielded 12,004 potentially relevant citations in accordance with the search strategy. A total of 11,886 articles were screened after 118 duplicates of the same articles included in different databases were removed. According to the inclusion criteria, 11,731 articles were excluded on the basis of the titles and abstracts. These studies were primarily excluded because they were not RCTs. After a full text reading of 155 studies, 82 articles were excluded for the following reasons: the participants did not meet the inclusion criteria (n = 70), duplication (n = 2), no control group (n = 3), the intervention included another Chinese herbal formula (n = 5), and no data for extraction (n = 2). Ultimately, 73 studies were included for analysis.^21–93^

### Study Characteristics

The characteristics of the 73 included trials are summarized in Table 1 . Of the 82 TCPMs, definite or possible RCTs and CCTs for 17 TCPMs were identified. For the remaining 67 TCPMs, we only identified case reports, case series, and uncontrolled observational studies. Seventy-three trials regarding 17 TCPMs were included, of which 63 and 10 trials were definite or possible RCTs and CCTs, respectively. All trials were conducted in China and published in Chinese journals (from 1994 to 2013).

**TABLE 1:**
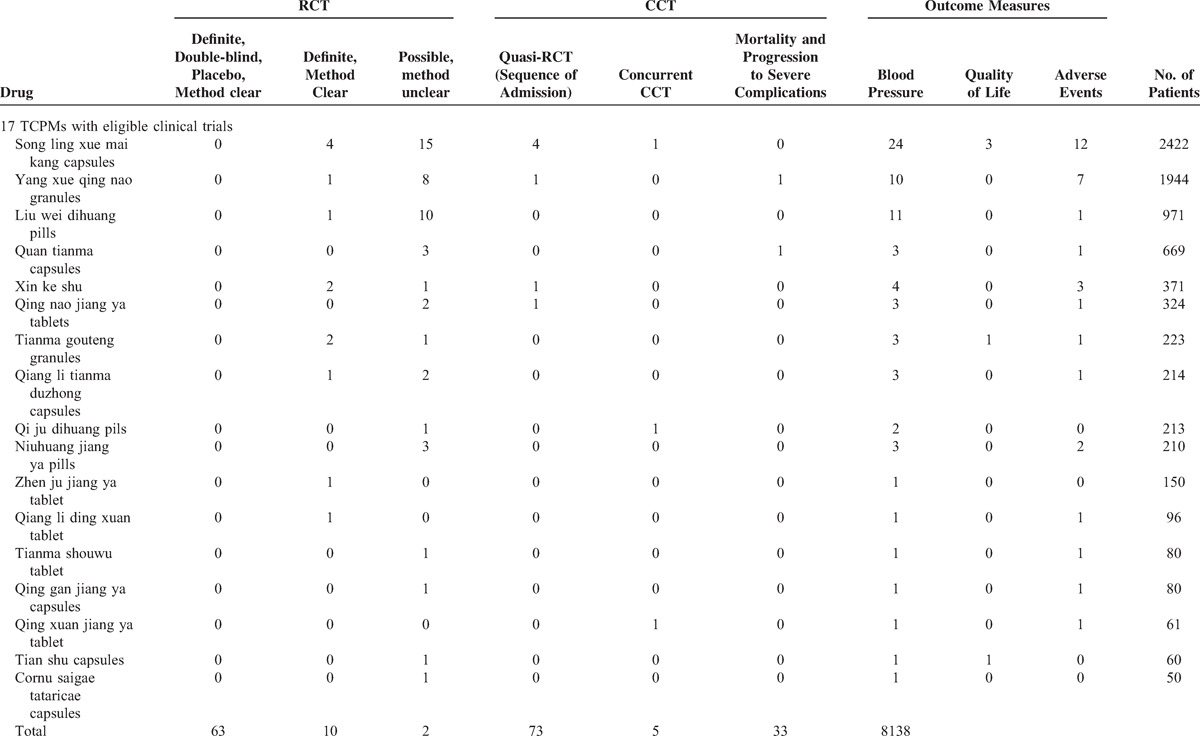
Basic Characteristics of the Trials and Patients Regarding TCPMs for EH

**TABLE 1 (Continued):**
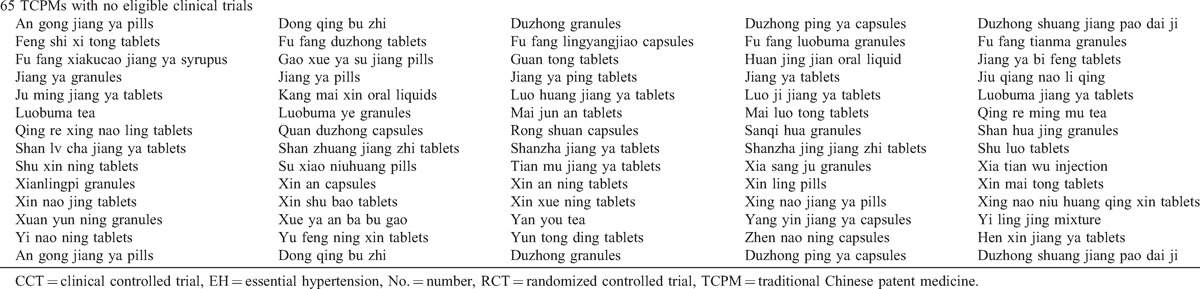
Basic Characteristics of the Trials and Patients Regarding TCPMs for EH

The RCTs included 8138 participants with EH. The age of the hypertensive patients ranged from 23 to 88 years. Standard Western medicine diagnostic criteria for EH was applied in all included trials. Additionally, many trials used TCM diagnostic criteria according to TCM theory. The formulations of the TCPMs included capsules, granules, pills, oral liquid, and tablets. Components of the 17 included TCPMs were depicted in Table 2 .^21–93^ All 73 trials were single-center trials and had parallel designs that compared TPAD in a treatment group versus antihypertensive drugs in a control group. The patients in the treatment group were treated with the same type and dosage of antihypertensive drugs under the same standard in the control group. The total treatment duration ranged from 2 weeks to 1 year.

**TABLE 2:**
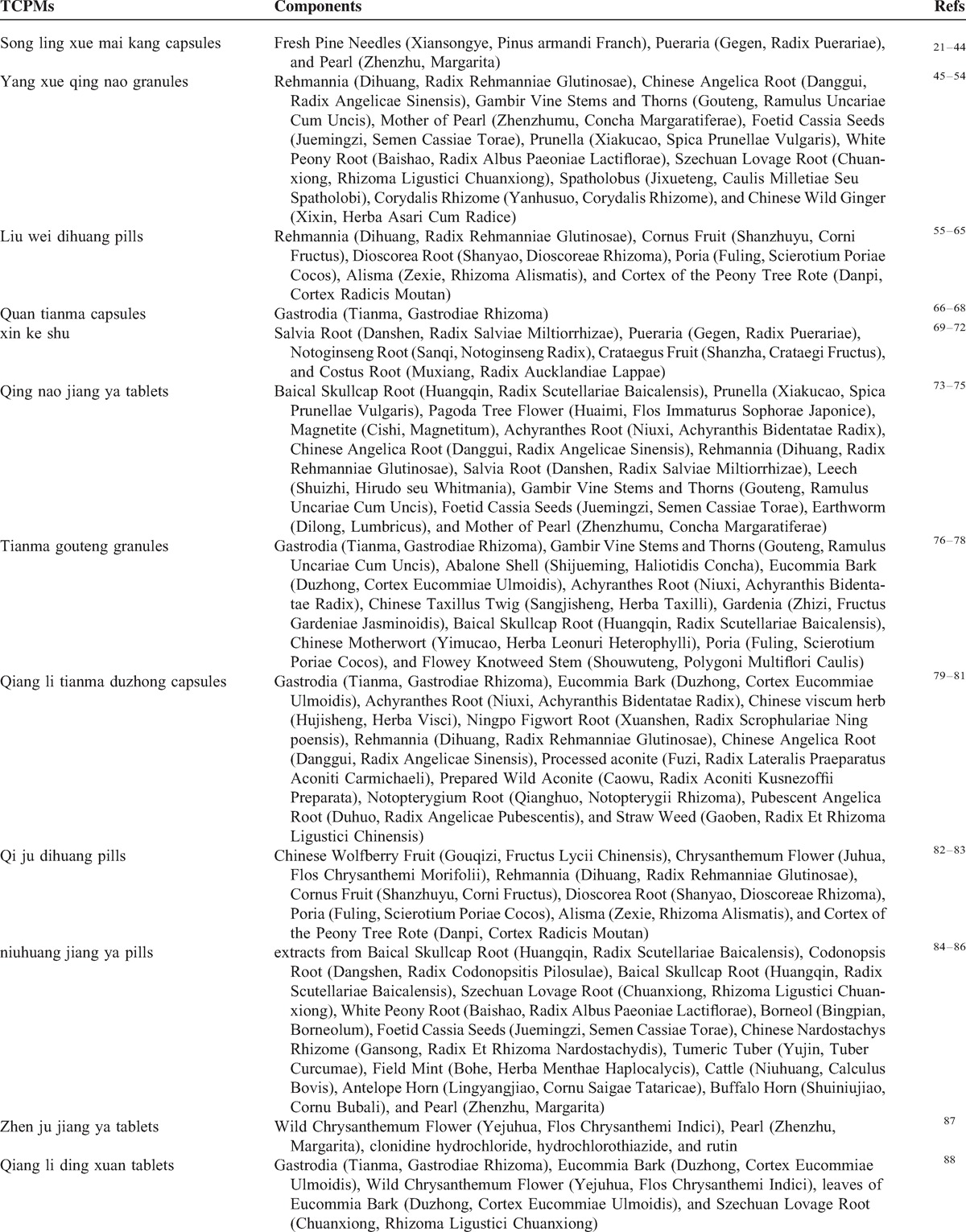
Components of the Included TCPMs

**TABLE 2 (Continued):**
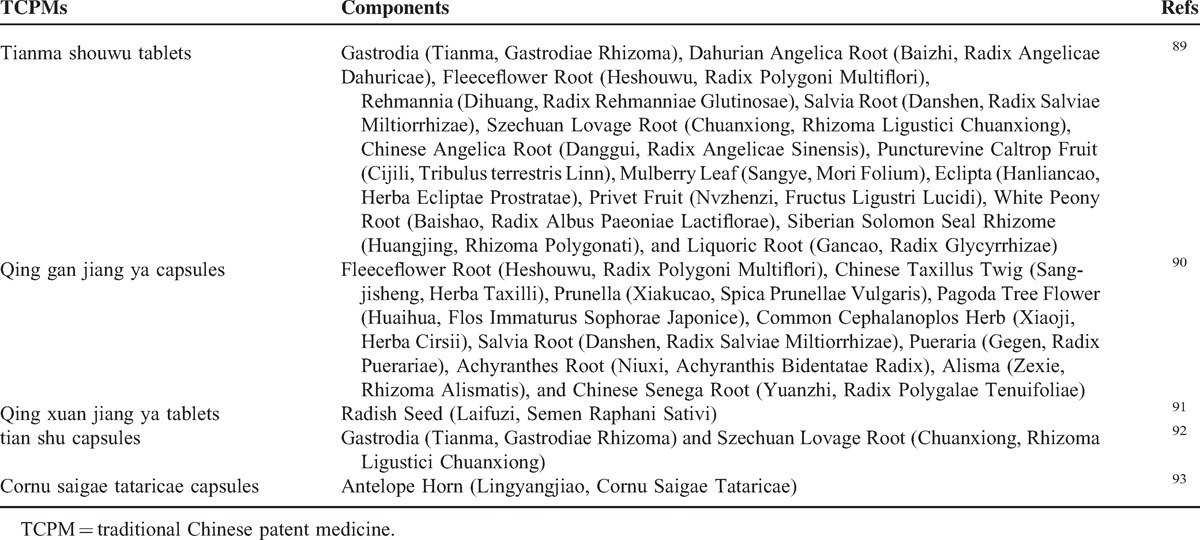
Components of the Included TCPMs

Two trials reported mortality and progression to severe complications after a 1-year follow-up.^46,66^ BP was assessed in all included trials. The treatment durations of 5 trials was >6 months.^42,46,50,66,89^ The QOL was reported in 5 trials,^33,36,42,76,92^ of which 4 trials^33,36,42,76^ used the Croog Scale,^94^ and 1 trial^92^ used the SF-36 Scale.^95^ The AEs of 33 trials have been described in detail.^21–23,28–30,33,35,38–40,43,45–50,53,60,68–71,74,77,79,84,85,88–91^

### Risk of Bias in Individual Studies

The risk of bias in the included trials was assessed as unclear and high according to the predefined Cochrane criteria. No randomized, double-blind, and placebo-controlled trial was identified. Only 4 trials on Song ling xue mai kang capsules,^21,23,28,44^ 1 trial on Yang xue qing nao granules,^51^ 1 trial on Liu wei dihuang pills,^65^ 2 trials on Xin ke shu,^70,71^ 2 trials on Tianma gouteng granules,^76,77^ 1 trial on Qiang li tianma duzhong capsules,^80^ 1 trial on Zhen ju jiang ya tablets,^87^ and 1 trial on Qiang li ding xuan tablets^88^ reported methods for randomization. The remaining 50 trials simply declared that patients with EH were randomized into treatment and control groups without a description of the specific method. No detailed information was provided to judge whether the study was conducted properly. One trial reported the blinding of participants and personnel.^28^ Allocation concealment and the blinding of the outcome assessment were not mentioned in all included trials. Six trials reported drop-outs or withdrawals.^21,37,46,56,60,79^ A pre-trial estimation of sample size was not reported in all trials. We attempted to contact the corresponding authors via telephone, fax, and email for inadequate information; however, most studies did not provide detailed contact information, and no additional information could be obtained to date.

### Primary Outcomes

#### Mortality and Progression to Severe Complications

The effects of TCPM on mortality and the progression to severe complications were only reported in 2 trials.^46,66^ One trial on Yang xue qing nao granules (130 patients) assessed the incidence of mortality and progression to severe complications at 1 year after treatment compared with antihypertensive drugs (with no detailed information regarding the classification or dosage).^46^ No deaths or severe complications occurred in the Yang xue qing nao granules group. However, 5 cases of coronary heart disease and 2 cases of transient ischemic attack were identified in the antihypertensive drugs group. A meta-analysis indicated that there was no significant difference between Yang xue qing nao granules and antihypertensive drugs regarding the incidence of coronary heart disease (RR: 0.09; 95% CI: 0.00–1.60; *P* = 0.10) or a transient ischemic attack (RR: 0.20; 95% CI: 0.01–4.25; *P* = 0.30).

Another trial also evaluated the effects of quan tianma capsules (500 patients) on the incidence of mortality and the progression to severe complications compared with nifedipine sustained-release tablets (10 mg, bid).^66^ The meta-analysis indicated that there was no significant difference between quan tianma capsules and nifedipine sustained-release tablets for the incidence of coronary heart disease (RR: 0.84; 95% CI: 0.54–1.29; *P* = 0.43), myocardial infarction (RR: 0.57; 95% CI: 0.24–1.36; *P* = 0.21), heart failure (RR: 0.42; 95% CI: 0.11–1.57; *P* = 0.20), cerebral infarction (RR: 1.04; 95% CI: 0.66–1.62; *P* = 0.87), cerebral hemorrhage (RR: 0.43; 95% CI: 0.13–1.44; *P* = 0.17), transient ischemic attack (RR: 1.15; 95% CI: 0.76–1.72; *P* = 0.51), renal failure (RR: 0.91; 95% CI: 0.42–1.96; *P* = 0.81), retinopathy (RR: 1.06; 95% CI: 0.49–2.32; *P* = 0.88), or death (RR: 0.26; 95% CI: 0.06–1.04; *P* = 0.06).

#### AEs

**FIGURE 2:**
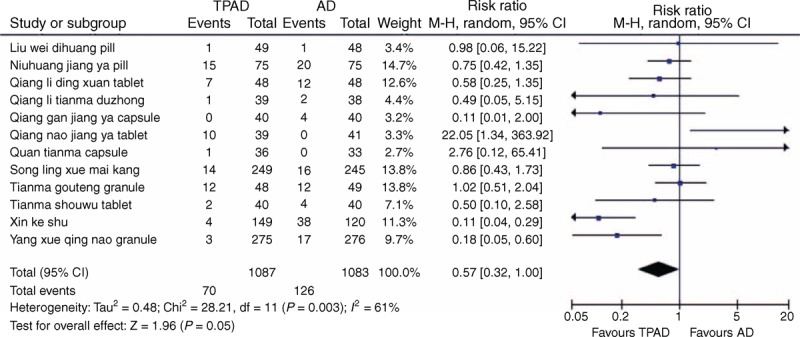
Forest plot of the reported AEs with 13 TCPMs. AD = antihypertensive drugs, AEs = adverse events, TCPM = traditional Chinese patent medicine.

AE monitoring was reported in 33 trials on 13 TCPMs,^21–23,28–30,33,35,38–40,43,45–50,53,60,68–71,74,77,79,84,85,88–91^ whereas it is not mentioned in the remaining 40 trials. The 13 included TCPMs were Liu wei dihuang pills, Niuhuang jiang ya pills, Qiang li ding xuan tablets, Qiang li tianma duzhong capsules, Qing gan jiang ya capsules, Qing nao jiang ya tablets, Qing xuan jiang ya tablets, Quan tianma capsules, Song ling xue mai kang capsules, Tianma gouteng granules, Tianma shouwu tablets, Xin ke shu, and Yang xue qing nao granules. Of the 33 trials, no AEs were reported in 10 trials and with Qing xuan jiang ya tablets. The AEs of the other 12 TCPMs included orthostatic hypotension, headache, dizziness, facial flushing, palpitations, nasal congestion, drowsiness, rash, pruritus, dry mouth, cough, nausea, abdominal discomfort, diarrhea, constipation, and ankle edema. No serious AEs were identified, and all AEs disappeared without special treatment. There were 1087 patients in the TPAD group and 1083 patients in the antihypertensive drugs group. Because significant heterogeneity among the trials on every TCPM existed (chi-square = 28.21, *P* = 0.003; *I*^2^ *=* 61%), a random-effects model was applied. A meta-analysis of all 12 TCPMs indicated that there was no significant difference regarding the AEs between TPAD and antihypertensive drugs (RR: 0.57; 95% CI: 0.32–1.00; *P* = 0.05), with the exception of Xin ke shu (RR: 0.11; 95% CI: 0.04–0.29; *P* < 0.0001), Yang xue qing nao granules (RR: 0.18; 95% CI: 0.05–0.60; *P* = 0.005), and Qing nao jiang ya tablets (RR: 22.05; 95% CI: 1.34–363.98; *P* = 0.03) (Figure 2).

### Secondary Outcomes

#### BP

**FIGURE 3:**
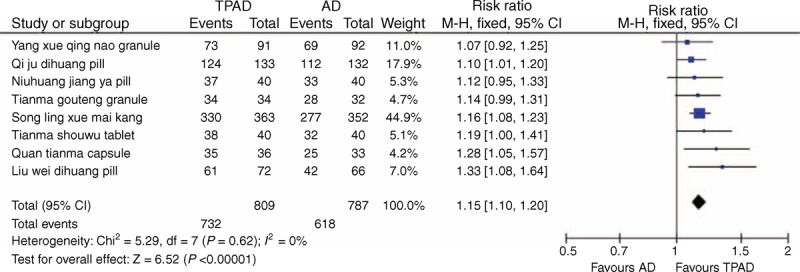
Forest plot of the trials that compared TPAD with antihypertensive drugs for BP. AD = antihypertensive drugs, BP = blood pressure, TPAD = traditional Chinese patent medicine plus antihypertensive drugs.

All 73 included trials evaluated the effects of TCPMs on BP in individuals with EH. Fourteen trials used the categorical BP to evaluate the efficacy of 8 TCPMs, the evaluation criteria of which were authoritatively recommended by the CFDA and the Guidelines of Clinical Research of New Drugs of Traditional Chinese Medicine. The effects were described as follows: “significant improvement” (DBP decreased by 10 mmHg and reached the normal range or DBP did not return to normal but decreased by more than 20 mmHg), “improvement” (DBP decreased by <10 mmHg but reached the normal range, DBP decreased by 10–19 mmHg but did not reach the normal range, or SBP decreased by >30 mmHg), and “no improvement” (did not meet the previously discussed standards).^96^ To permit at least some overall analysis, these outcomes were converted into dichotomous data. Therefore, we grouped together “significant improvement” and “improvement” as “effective” and “no improvement” as “ineffective.” The 8 TCPMs that were included were Liu wei dihuang pills, Niuhuang jiang ya pills, Qi ju dihuang pills, Quan tianma capsules, Song ling xue mai kang capsules, Tianma gouteng granules, Tianma shouwu tablets, and Yang xue qing nao granules. There were 809 patients in the TPAD group and 787 individuals in the antihypertensive drugs group. Fixed-effects model was used because no significant heterogeneity of the trials was identified (chi-square = 5.29, *P* = 0.62; *I*^2^ *=* 0%). A meta-analysis of the 14 trials on the 8 TCPMs identified a significant BP-lowering effect of TPAD (RR: 1.15; 95% CI: 1.10–1.20; *P* < 0.00001) compared with antihypertensive drugs alone (Figure 3).

Fifty-nine trials used continuous BP to evaluate the treatment effects of 16 TCPMs in hypertensive patients, with the exception of Tianma shouwu tablets. The combined results suggested that 13 TCPMs (13/16, 81.25%) used as complementary therapy to antihypertensive drugs significantly decreased SBP by 3.94–13.50 mmHg; however, the other 3 TCPMs (3/16, 18.75%) decreased SBP by 1.00–5.00 mmHg, with no significant difference compared with antihypertensive drugs alone. The meta-analysis also indicated that 9 TCPMs (9/16, 56.25%) used as complementary therapy to antihypertensive drugs significantly decreased DBP by 2.28–11.25 mmHg; however, the other 7 TCPMs (7/16, 43.75%) decreased DBP by -1.00–9.41 mmHg with no significant difference compared with antihypertensive drugs alone. The detailed effect sizes of TPAD compared with antihypertensive drugs are summarized in Table 3. Two TCPMs, Song ling xue mai kang capsules (854 vs 835) and Yang xue qing nao granules (882 *vs.* 879), had more studies and >1000 patients, respectively.

**TABLE 3:**
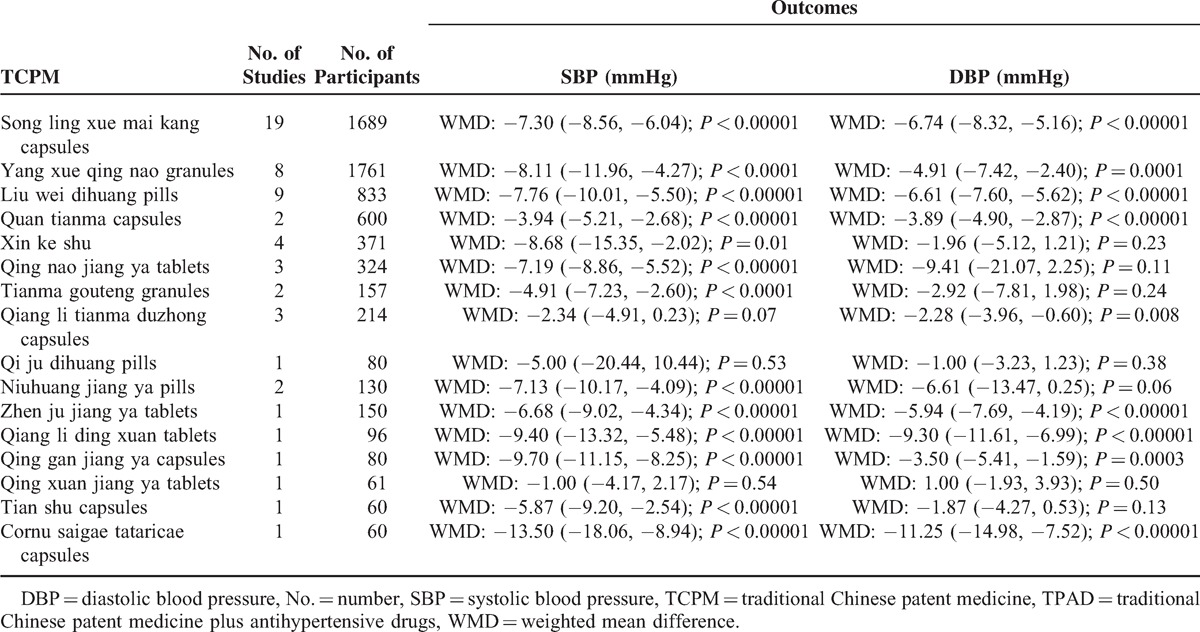
Effect sizes of TPAD Compared with Antihypertensive Drugs

#### Quality of Life

Both the Croog and SF-36 scales were used to assess the QOL in 5 trials regarding Song ling xue mai kang capsules,^33,36,42^ Tianma gouteng granules,^76^ and Tian shu capsules.^92^ All 5 trials reported a significant improvement in the QOL of the TPAD group compared with the antihypertensive drug group at the end of treatment. One trial on Song ling xue mai kang capsules^33^ demonstrated that the Croog Scale increased with the total score (WMD = 26.10; 95% CI: 25.88–26.32; *P* < 0.00001).

The other 2 trials on song ling xue mai kang capsules^36,42^ all reported a significant improvement in the sense of well-being (WMD = 7.70; 95% CI: 7.54–7.87; *P* < 0.00001), physical symptoms (WMD = 2.56; 95% CI: 2.28–2.84; *P* < 0.00001), work performance and satisfaction (WMD = 2.54; 95% CI: 2.37–2.72; *P* < 0.00001), and life satisfaction (WMD = 3.48; 95% CI: 3.28–3.68; *P* < 0.00001) compared with the antihypertensive drug group. One trial on Tianma gouteng granules^76^ demonstrated a significant improvement on the Croog Scale (WMD = 13.93; 95% CI: 8.10–19.76; *P* < 0.00001). One trial on tian shu capsules^92^ exhibited a significant reduction in the total score of the SF-36 Scale (WMD = −27.56; 95% CI: -28.67 to -26.45; *P* < 0.00001).

## DISCUSSION

### Summary of Evidence

The previous decade has witnessed an unprecedented expansion of the field of antihypertensive treatment and drug discovery and development. Thus, Western medicine became the dominant medical treatment worldwide. However, it has been increasingly realized that Western medicine may sometimes fail to treat cardiovascular diseases (CVDs), especially in patients with a history of conventional medicine-related AEs, whereas CAM is becoming increasingly popular and frequently used among patients with CVDs.^97,98^ As a result of the different theoretical systems and control methods, Western physicians often find CAM difficult to understand. TCM has continued to hold an important position in primary healthcare both in China and other Asian countries.^99^ Additionally, there is one important characteristic of China's national medical system that facilitates CAM, that is, integrative medicine, which combines TCM and Western medicine together in the clinic and is responsible for health care.^100,101^ Therefore, as an adjunctive treatment to antihypertensive drugs, TCPM is a popular natural product for EH.^102^ Because of its health-enhancing qualities, TCPM has been developed and used in China for >30 years. Currently, there are 82 TCPMs approved by the CFDA for the treatment of EH. However, few TCPMs have been evaluated regarding their effectiveness and safety according to current scientific standards; furthermore, few relevant articles on TCPMs for EH have been published in English-language medical journals. There is a lack of studies that assess the majority of the commonly used TCPMs for EH, which has limited their clinical use and weakened the credibility of the study of TCPM internationally. In this article, although many TCPMs did not meet our selection criteria, the findings provided the current status of the clinical evidence regarding the approved TCPMs for EH in China. The methodological quality of most included trials was evaluated as generally “poor” because of inadequate reporting of their methods in detail. However, we did not arbitrarily exclude them because we sought to report on the overall quality. As the first systematic review, this study aimed to assess the quantity, quality, and overall strength of the evidence regarding the most commonly used and government-recommended TCPMs for EH.

There are several strengths in this study. Previous studies have demonstrated that antihypertensive medicine treatment is associated with a substantial reduction of cardiovascular events and mortality.^103^ There is the possibility that TCPM could, as a complementary therapy to antihypertensive drugs, more significantly lower the incidence rate of major cardiovascular events. In this review, the first finding was the evidence regarding the efficacy of the evaluated TCPMs on primary outcomes. There was inadequate reporting of primary outcome measures. Only 2 trials on Yang xue qing nao granules and Quan tianma capsules assessed the long-term outcomes of mortality and progression to severe complications after treatment. No significant difference between the treatment and control groups was identified. Therefore, no convincing evidence of the beneficial effects that support the routine use of TCPM as an adjunctive therapy for EH could be drawn because of the very limited numbers of RCTs and the patients in these 2 trials. Nonetheless, the findings demonstrated that long-term outcomes of TCPM could be evaluated with RCTs.

Another valuable finding of this review was the estimation on the incidence of AEs. There was a lack of knowledge regarding the significance of reporting the frequency of AEs in the RCTs. TCPM appeared to be free of major AEs. The reported AEs were not severe and required no additional special treatment. Although the aggregated results indicated that there was no difference regarding the AEs between the 2 groups, the safety of TCPMs must still be rigorously monitored and appropriately reported in future clinical trials.

**FIGURE 4:**
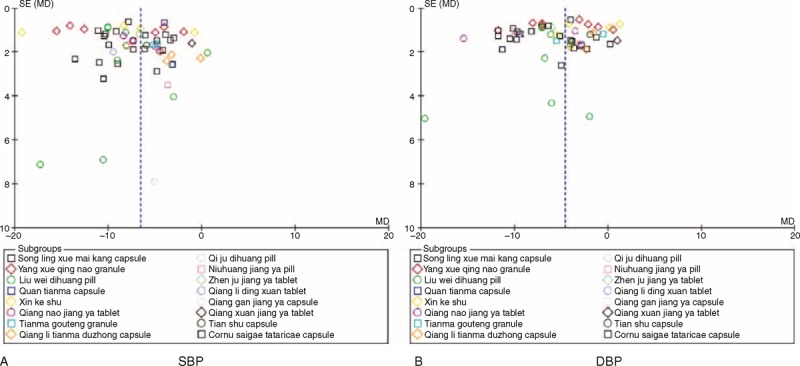
Funnel plot of the trials that compared TPAD with antihypertensive drugs. (a) SBP and (b) DBP. DBP = diastolic blood pressure, SBP = systolic blood pressure, TPAD = traditional Chinese patent medicine plus antihypertensive drugs.

The most striking finding in this systematic review was the estimation of the therapeutic effects of TCPM on secondary outcomes. We also evaluated the effectiveness of TCPM in lowering BP in individuals with EH. The BP outcomes were reported in all included trials. The meta-analysis indicated that 13 TCPMs (81.25%) possess a better BP-lowering effect as complementary therapies, with a SBP decrease of 3.94 to 13.50 mmHg and a DBP decrease of 2.28 to 11.25 mmHg. For example, a significant BP-lowering effect of the most commonly used 2 TCPMs, song Ling xue mai kang capsules (SBP decreased by 7.30 mmHg and DBP decreased by 6.74 mmHg) and Yang xue qing nao granules (SBP decreased by 8.11 mmHg and DBP decreased by 4.91 mmHg), was identified. However, confirmation regarding BP was limited because of the poor methodological quality, the lack of placebo-controlled trials, and the significant heterogeneity of the included trials (Figure 4). Therefore, more evidence is required to confirm these conclusions. If the beneficial effects on BP were confirmed by rigorously designed trials with high methodological quality, it could lead to the identification and use of many valuable treatments for EH.

The fourth finding was the evaluation of the effects of TCPM on QOL. In this review, the data regarding QOL were insufficient. Only 5 trials on 3 TCPMs (Song ling xue mai kang capsules, Tianma gouteng granules, and Tian shu capsules) assessed the QOL in hypertensive patients using the Croog and SF-36 scales. Although the QOL in the treatment groups was generally higher than in the control groups for 3 TCPMs, more rigorously designed RCTs are warranted to confirm these results.

## LIMITATIONS

Our study has several limitations. First, all included trials were of poor methodological quality. In our review, in fact, it was impossible to identify well-designed trials to evaluate the efficacy of TCPM for the management of EH. All the included trials were shown to have a high or unclear risk of bias due to design, reporting, and methodology. Inadequate reports of the study design, allocation sequence, allocation concealment, blinding, intention-to-treat analysis, and drop outs were identified in the majority of the trials. There were no definite randomized, double-blind, placebo-controlled trials with clear methodologies. Only 10 trials reported methods for randomization. Allocation concealment was not mentioned in any of the trials. Therefore, the “alleged” trials may not actually represent real RCTs, which could weaken the strength of the clinical evidence and lead to a potential selection bias. No trials were double-blinded. Only 1 trial reported the blinding of participants and personnel. Blinding of the outcome assessment was not mentioned. This would directly lead to performance and detection biases. Additionally, there was a lack of placebo control use. All trials included in the review used an “A + B versus B” design, in which patients with EH are randomized to receive treatment (A) combined with treatment (B) versus treatment (B) alone. This type of add-on design is quite popular in TCM studies in which TCM therapy is added to conventional therapy. Nevertheless, without a rigorous control for a placebo effect, this approach may exaggerate the effects of TCPM and is prone to generate false-positive results and significant systemic errors in the assessment of outcomes. We understood that such bias is a common problem in all TCM studies. Perhaps certain physical features associated with TCPMs, such as aromas, colors, and appearances, limited clinical placebo use. Unfortunately, none of the RCTs utilized a placebo control in the trials. Only a few trials reported drop-outs or withdrawals. Most of the trials did not report details of the intention-to-treat analysis. No trials had a pre-trial estimation of sample size. Thus, whether a sample size met the requirements of a given trial remains unknown.

Second, publication bias is another major cause of bias that should also be considered. We have made efforts to avoid language and location biases; however, only trials conducted in China and published in Chinese could be included for further analysis after comprehensive searches. Therefore, we cannot completely rule out the potential publication bias.

Furthermore, other systematic reviews regarding CAM have encountered similar problems.^104^ Recently, through huge investments in scientific research and the economical exploitation of TCM, the Chinese government has undertaken enormous efforts to modernize TCM and aims to prove the efficacy of TCM according to international standards. However, in our review, only 1 of the 73 selected articles declared their sources of funding.^51^

Overall, the results of the trials included in this systematic review are likely to be biased by many factors because of the poor methodological qualities of these studies. The reported beneficial effect of TCPM in lowering BP in hypertensive patients should be interpreted cautiously. Additionally, 2 TCPMs, Song ling xue mai kang capsules and Yang xue qing nao granules, had more studies and greater patient numbers (>1000 patients) and may be worthy of further research.

## CONCLUSIONS

In summary, this systematic review provided the first limited piece of clinical evidence regarding the effectiveness of TCPMs as a complementary therapy in improving mortality, the progression to severe complications, AEs, BP, and QOL. According to the Oxford Center for Evidence-Based Medicine 2011 Levels of Evidence, the usage of TCPMs for EH was supported by evidence of class level III. However, because of the negative results on primary outcomes, insufficient clinical data, poor methodological quality, small sample sizes, limited numbers of each trial, and the high heterogeneity of the studies, the current evidence is insufficient to support a recommendation for wide TCPM use.

Therefore, future studies should overcome the limitations of the trials included in this systematic review. The following methodological issues should be cautiously addressed: adequate generation of allocation sequences and the concealment of allocation; appropriate methods of double-blinding (blinding of participants and personnel and blinding of outcome assessment); wider use of placebo controls; strict reporting regarding drop-outs and the use of intention-to-treat analysis; a specifically focus on mortality and cardiovascular events with long-term follow-up; and comprehensive reporting of the trials according to the recommendations of the CONSORT Statement.^105,106^ We also suggest that Song ling xue mai kang capsules and Yang xue qing nao granules, which were evaluated to be with the evidence of class level III, should be prioritized for further research. We hope that this systematic review will pave the way for the generation of evidence-based TCPMs for the treatment of EH.
